# Prognostic Factors in Squamous Cell Carcinoma of the Maxillary Gingiva and Hard Palate: A Retrospective Analysis From a Single Institution

**DOI:** 10.7759/cureus.78487

**Published:** 2025-02-04

**Authors:** Kei-ichiro Miura, Mitsunobu Otsuru, Keisuke Omori, Tomofumi Naruse, Masahiro Umeda, Tomohiro Yamada

**Affiliations:** 1 Department of Oral and Maxillofacial Surgery, Nagasaki University Graduate School of Biomedical Sciences, Nagasaki, JPN; 2 Department of Oral and Maxillofacial Surgery, Kanagawa Dental University, Yokosuka, JPN

**Keywords:** extranodal extension, maxillary gingiva, metastasis, prognosis, squamous cell carcinoma

## Abstract

*Background*:* *The maxilla is surrounded by complex anatomical structures, including the nasal cavity, maxillary sinus, zygomatic process, cranial base, mandible, and masticatory muscle space. Therefore, when maxillary squamous cell carcinoma (SCC) is close to or invades these structures, determining clear surgical margins becomes challenging, making complete resection difficult.This study aimed to evaluate factors related to primary recurrence, cervical lymph node recurrence, distant metastasis, and prognosis of primary maxillary SCC at the Department of Oral and Maxillofacial Surgery, Nagasaki University Hospital.

*Methodology*:* *Patients with SCC of the maxillary gingiva or hard palate treated at a single institute between 2008 and 2022 were reviewed. Age, sex, performance status, primary site, T stage, N stage, pathological N stage, histological differentiation, mode of invasion, lymphatic invasion, vascular invasion, perineural invasion, margin status, extranodal extension, treatment method, and outcome were reviewed. Factors related to survival and local recurrence were analyzed by Cox regression.

*Results*:* *A total of 61 patients were enrolled. The five-year overall survival (OS) and disease-specific survival (DSS) rates were 74.4% and 83.3%, respectively. Univariate analysis revealed that performance status, T stage, mode of invasion, pathological N stage, lymphatic invasion, vascular invasion, perineural invasion, extranodal extension, and local recurrence were significantly associated with OS and DSS. Local recurrence was observed in 14 of 61 patients (23.0%), all of which had T4 tumors. Finally, 12 patients had uncontrolled primary tumors, while only two had uncontrolled cervical metastases. Margin status did not significantly affect survival and local recurrence.

*Conclusions*: While uncontrolled disease was observed in 14 cT4 cases, no local recurrence occurred in cT1-T3 cases. Histological factors, not margin status, were key prognostic indicators, suggesting the need to re-evaluate treatment strategies for T4 and high-grade tumors.

## Introduction

Oral cancer, though a significant global health concern, accounts for only 2% of all cancer cases. Mortality related to oral cancer contributes to 1.8% of cancer-related deaths, indicating its relatively lower incidence compared to other malignancies. However, the distribution of oral cancer is not uniform worldwide. In certain regions, particularly Southeast Asia, the prevalence is notably higher due to cultural and lifestyle factors such as chewing tobacco, betel nut consumption, smoking, and alcohol use [1]. Outside these high-risk areas, oral cancer is considered a rare malignancy.

Within the spectrum of oral cancers, maxillary gingival squamous cell carcinoma (SCC) is even more uncommon, constituting only 6%-7% of all oral SCCs [2]. This rarity poses several clinical and research challenges, including limited available data, difficulty in establishing standardized treatment protocols, and a lack of large-scale studies assessing prognostic factors and treatment outcomes. The scarcity of cases makes it challenging to conduct randomized clinical trials, leading to treatment decisions that often rely on retrospective analyses and expert consensus rather than high-level evidence.

One of the most significant challenges in treating maxillary gingival SCC is the anatomical complexity of the maxilla. This region is closely surrounded by critical structures, including the nasal cavity, maxillary sinus, zygomatic process, cranial base, mandible, and masticatory muscle space. Due to these intricate anatomical relationships, achieving adequate surgical margins is particularly difficult when the tumor is close to or has infiltrated these structures. Complete resection, which is essential for reducing recurrence, may not always be feasible without significantly compromising function. Consequently, local recurrence rates remain a major concern, often necessitating additional interventions such as adjuvant radiotherapy (RT) or chemotherapy.

Despite advances in oncologic surgery and RT, several key clinical questions regarding the optimal treatment approach for maxillary gingival SCC remain unresolved. One major issue is whether surgery alone is sufficient or if chemoradiotherapy (CRT), particularly intra-arterial chemotherapy infusion, should be incorporated into the treatment strategy. Some studies have suggested that intra-arterial chemotherapy, which delivers high-dose anticancer drugs directly to the tumor site, may improve local tumor control [3]. However, high-quality evidence supporting its superiority over conventional treatment remains scarce.

Another significant concern is the determination of appropriate resection margins for primary tumor excision. Unlike oral cancers in other regions, maxillary tumors often extend into bony structures, making it difficult to define an ideal resection boundary. Insufficient margins may lead to higher recurrence rates, while overly aggressive resections could result in severe functional impairments, including difficulties in chewing, speaking, and facial aesthetics.

The role of elective neck dissection (END) in clinically node-negative (N0) cases also remains controversial. While some studies have reported occult metastases in 15%-30% of cases, suggesting a potential benefit of END, others argue that routine END may lead to overtreatment and unnecessary complications [4,5]. The challenge lies in identifying which patients truly require END to reduce recurrence risk while avoiding unnecessary surgical morbidity.

Another complex issue is the approach to tumors that have invaded the masticatory muscle space [6,7,8]. When tumors progress posteriorly into this region, surgical resection becomes highly challenging. Removing the masticatory muscle can lead to severe functional impairments, so the decision to perform an aggressive resection versus more conservative management remains a subject of debate.

Given these unresolved clinical questions and the rarity of maxillary gingival SCC, this study aims to retrospectively analyze cases from our institution to provide valuable insights into the disease's prognosis and treatment strategies. Specifically, the study seeks to characterize patient demographics and clinical features, evaluate recurrence patterns (primary tumor recurrence, cervical lymph node recurrence, and distant metastasis), and identify prognostic factors that influence survival outcomes.

## Materials and methods

Inclusion and exclusion criteria

This study was designed to include patients who met specific eligibility criteria to ensure a well-defined and homogenous study population. The inclusion criteria were established to select patients with primary maxillary SCC who underwent surgical treatment at the Department of Oral and Maxillofacial Surgery at Nagasaki University Hospital between July 2008 and July 2022.

To be eligible for inclusion, patients had to meet all of the following criteria. First, they had to be 18 years or older at the time of diagnosis. Second, they must have visited the Department of Oral and Maxillofacial Surgery at Nagasaki University Hospital during the specified study period and received a histopathological diagnosis of primary maxillary SCC through biopsy, followed by surgical treatment. Additionally, only patients with no prior history of primary maxillary SCC were included, ensuring that the study focused solely on newly diagnosed cases.

Furthermore, patients were only considered for inclusion if they had no previous history of treatment such as chemotherapy, RT, molecular-targeted therapy, or immune checkpoint inhibitors for their primary tumor, cervical region, or any other cancer types. This criterion was established to prevent the influence of prior treatments on the study outcomes. Another critical requirement was that patients had not received any preoperative treatment for primary maxillary SCC, ensuring that the tumor characteristics at the time of diagnosis were not altered by prior medical interventions. Finally, regular postoperative follow-up was required, allowing for a thorough assessment of recurrence, metastasis, and long-term prognosis.

On the other hand, exclusion criteria were defined to eliminate cases that could confound the study results or introduce variability in tumor pathology. Patients were excluded if they had active multiple primary cancers, as this could significantly impact treatment decisions and prognosis. Additionally, patients whose tumors were not histologically confirmed as SCC were excluded, ensuring that only SCC cases were analyzed. Another exclusion criterion was applied to patients whose histopathological findings suggested an oral potentially malignant disorders rather than a true carcinoma, as their clinical course and treatment requirements differ from actual malignant tumors. Finally, any patient deemed unsuitable for inclusion by the attending physician was also excluded, allowing clinical judgment to guide the selection process in cases where eligibility was uncertain.

By implementing these strict inclusion and exclusion criteria, this study aimed to ensure the reliability of its findings, focusing exclusively on primary maxillary squamous cell carcinoma while minimizing confounding factors.

Variables assessed

In this study, data were systematically collected from electronic medical records to ensure a comprehensive analysis of patients diagnosed with primary maxillary SCC. The information gathered encompassed demographic, clinical, pathological, and prognostic factors, which were essential for evaluating survival outcomes and recurrence patterns.

The patient background included age at diagnosis, sex, Eastern Cooperative Oncology Group-Performance Status (ECOG-PS) [9], and primary tumor site (maxillary gingiva or hard palate). Additionally, the date of the first visit to the oral and maxillofacial surgery department and the treatment completion date (either the surgery date or the completion of postoperative adjuvant therapy) were recorded.

Tumor classification was conducted according to the 8th edition of the UICC staging system, which included cT classification, cN classification, and pN classification [10]. Histopathological findings were also assessed, including histological differentiation (categorized as well-differentiated, moderately differentiated, or poorly differentiated) and mode of invasion using the Yamamoto-Kohama (YK) classification [11]. Other pathological factors analyzed included the presence of lymphatic invasion, vascular invasion, perineural invasion, and extranodal extension (ENE) of metastatic lymph nodes. The surgical margin status was also evaluated, with margins classified as *clear* if they were ≥5 mm and *involved/close* if they were <5 mm.

In addition, information on neck dissection was collected, specifying whether no neck dissection was performed, neck dissection was conducted initially, or a secondary neck dissection was performed.

To assess survival outcomes, five-year overall survival (OS) was calculated as the duration (in months) from the date of diagnosis to death, regardless of the cause. Similarly, five-year disease-specific survival (DSS) was determined based on the duration (in months) from the initial visit to death due to primary maxillary SCC.

Furthermore, local recurrence (local control) was evaluated by assessing the presence or absence of recurrence and measuring the time from initial treatment to local recurrence.

The YK classification defines tumor invasion patterns based on their degree of infiltration into surrounding tissues. YK1 tumors have a well-defined borderline, indicating minimal invasion. YK2 tumors exhibit a cord-like invasion pattern with a less distinct borderline. YK3 tumors consist of small groups of cells infiltrating the surrounding tissue, without a clearly defined boundary. YK4C tumors demonstrate diffuse invasion with a cord-like infiltration pattern, showing a higher degree of tissue penetration. Lastly, YK4D tumors display widespread diffuse invasion, representing the most aggressive form of infiltration.

Statistical analysis

Sex, ECOG-PS, site, cT stage, cN stage, pN stage, histological differentiation, mode of invasion, margin status, lymphatic invasion, vascular invasion, perineural invasion, and number of positive cases were presented as numerical values (percentages). Age was presented as the mean ± standard deviation (SD). Univariate Cox regression analyses were conducted for five-year OS, five-year DSS, and local control based on the clinical and pathological variables. In addition, some variables extracted as prognostic factors were illustrated using the Kaplan-Meier method and log-rank test. In the analysis of five-year OS and five-year DSS, patients who remained alive beyond five years were censored at five years to ensure consistency in survival estimation. All statistical analyses were performed using SPSS software (version 26.0, IBM Corp., Armonk, NY), and the level of significance was set at *P* < 0.05.

Ethics

This study conformed to the ethical guidelines of the Declaration of Helsinki and Ethical Guidelines for Medical and Health Research involving Human Subjects by the Ministry of Health, Labor, and Welfare of Japan. Ethical approval was obtained from the Institutional Review Board (IRB) of Nagasaki University Hospital (#23091105). As this was a retrospective study, patient-identifiable information was removed, and the research plan was published on the homepages of the Nagasaki University Hospital website with an opt-out option, according to IRB instructions.

## Results

Patient characteristics

Of the 469 patients who underwent surgery during the study period, 61 patients (26 males and 35 females; mean age, 72.6 years) were included in the study. Performance status (PS) was 0 in 40 (65.6%) patients, while PS 1-3 was observed in 21 (34.4%) patients. The primary site was the maxillary gingiva in 51 cases (83.6%) and the hard palate in 10 cases (16.4%). T stage was T1-3 in 28 cases (45.9%) and T4 in 33 cases (54.1%). The N stage was cN0 in 45 cases (73.8%) and cN1-3 in 16 cases (26.2%). The pathological N stage, including late metastasis, was pN0 in 43 cases (72.1%) and pN1-3 in 18 cases (27.9%). Overall, cases without metastasis accounted for approximately 70%. Regarding histological differentiation, almost all cases (93.4%) were well-differentiated SCC. The mode of invasion was YK1-3 in 51 cases (83.6%) and YK4 in 10 cases (16.4%). The surgical margin status was clear in 44 cases (72.1%) and close/positive in 17 cases (27.9%). The histological features of neck metastasis, including late metastases, were as follows: lymphatic invasion in 7 cases (11.5%), vascular invasion in 12 cases (19.7%), perineural invasion in 12 cases (19.7%), and ENE in nine cases (14.8%). Although up to three histologically positive lymph nodes were confirmed in most cases, five nodes were found in one case and six in two cases (Table 1).

**Table 1:** Patient characteristics. PS, performance status

Variables	Number of patients (%)/mean ± SD
Sex	
Male	26 (42.6)
Female	35 (57.4)
Age (years)	72.6 ± 12.0
PS	
0	40 (65.6)
1	15 (24.6)
2	5 (8.2)
3	1 (1.6)
Site	
Gingiva	51 (83.6)
Hard palate	10 (16.4)
T stage	
T1	12 (19.7)
T2	13 (21.3)
T3	3 (4.9)
T4a	31 (50.8)
T4b	2 (3.3)
N stage	
N0	45 (73.8)
N1	7 (11.5)
N2a	0 (0)
N2b	7 (11.5)
N2c	2 (3.2)
N3	0 (0)
pN	
(-)	43 (70.5)
(+)	18 (29.5)
Differentiation	
Well	57 (93.4)
Moderate	4 (6.6)
Poorly	0 (0)
Mode of invasion	
YK1	1 (1.6)
YK2	17 (27.9)
YK3	33 (54.1)
YK4c	8 (13.1)
YK4d	2 (3.3)
Margin status	
Clear	44 (72.1)
Close/positive	17 (27.9)
Lymphatic invasion	
pN(-)	43 (70.5)
(-)	11 (18.0)
(+)	7 (11.5)
Vascular invasion	
pN(-)	43 (70.5)
(-)	6 (9.8)
(+)	12 (19.7)
Perineural invasion	
pN(-)	43 (70.5)
(-)	6 (9.8)
(+)	12 (19.7)
Extranodal extension	
pN(-)	43 (70.5)
(-)	9 (14.8)
(+)	9 (14.8)
Number of positive node	
0	43 (70.5)
1	6 (9.8)
2	6 (9.8)
3	3 (4.9)
4	0 (0)
5	1 (1.6)
6	2 (3.3)

Treatment and clinical course

In our department, surgical resection without preoperative treatment is the first-choice treatment for oral cancer. In accordance with the National Comprehensive Cancer Network (NCCN) guidelines, the postoperative concurrent CRT regimen consists of three-weekly cisplatin (CDDP) combined with radiotherapy (RT). CDDP) at a dose of 100 mg/m² was administered on day 1, day 22, and day 43. RT was delivered using a fractionated method, with a dose of 2 Gy per session, administered once per day, five times per week, for a total cumulative dose of 66 Gy, in cases where pathological examination confirmed ENE of metastatic cervical lymph nodes, with or without positive surgical margins. For cases with positive surgical margins only, additional resection was considered whenever feasible. If the margins were confirmed to be negative after additional resection, RT was considered. However, postoperative adjuvant therapy may be highly invasive. Therefore, strict postoperative follow-up without any treatment or postoperative RT alone was considered according to both patients’ systemic condition and their aspiration, even if they are at high risk of recurrence. For the treatment of cervical metastatic lymph nodes, therapeutic neck dissection was performed for clinically metastatic positive lymph nodes, and strict follow-up was performed for clinically negative nodes without initial END. END was performed when free flap reconstruction was necessary due to the extensive resection area and the requirement for microvascular anastomosis.

**Figure 1:**
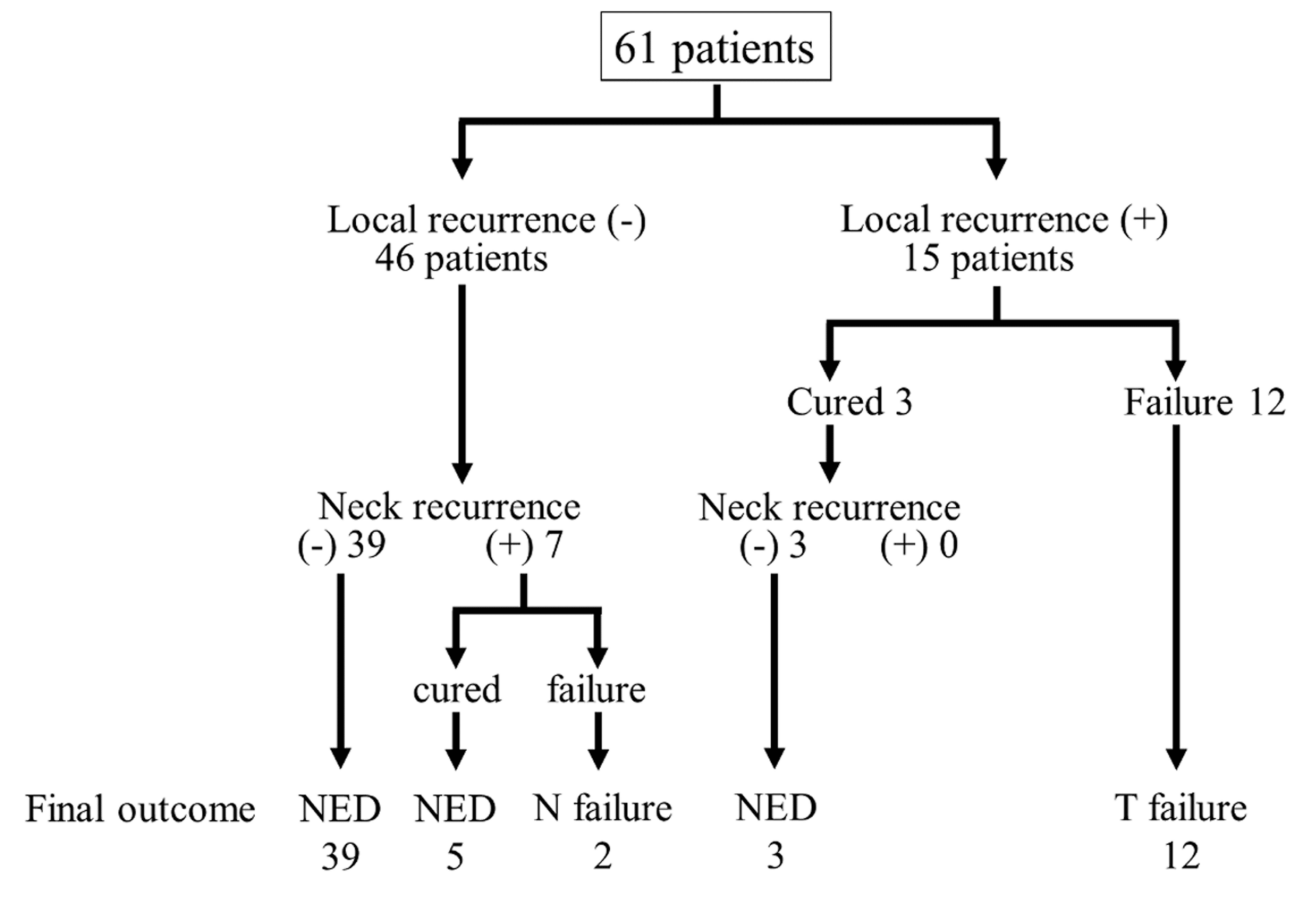
Treatment outcomes. Image credit: Masahiro Umeda. NED, no evidence of disease

The treatment outcomes are summarized in Figure 1.

Postoperative local recurrence was not observed in 46 patients, 39 of whom had no cervical metastases and were doing well. Seven of the 46 patients had initial or late cervical metastasis, and two of them had cervical recurrence that became uncontrolled. Postoperative local recurrence was observed in 15 patients, three of whom successfully controlled both the primary site and the neck. In contrast, 12 patients had local recurrence, and all patients succumbed to causes related to the primary tumor. In total, 47 patients (77.0%) had controlled tumors, 12 had uncontrolled primary tumors, and two had uncontrolled cervical metastases. No patient had distant metastases despite control of the primary site and neck.

Survival and factors related to OS and DSS

The factors associated with survival rates are summarized in Tables 2, 3.

**Table 2:** Factors related with OS (univariate analysis). ^*^P < 0.05. OS, overall survival; HR, hazard ratio; 95% CI, 95% confidence interval; PS, performance status; pN, pathological N; ENE, extranodal extension

Variable		*P*-value	HR	95% CI
Preoperative factor				
Age		0.571	0.985	0.937-1.037
Sex	Female/Male	0.329	0.556	0.171-1.807
PS	PS0/1/2/3	0.006*	2.623	1.324-5.195
Site	Gingiva/Hard palate	0.487	1.582	0.434-5.765
T stage	T1/2/3/4	0.031*	2.217	1.974-4.576
N stage	N0/1/2	0.637	0.822	0.364-1.856
Differentiation	Well/Moderate	0.976	1.931	0.134-7.950
Mode of invasion	YK1/2/3/4	0.012*	3.047	1.276-7.274
Postoperative findings				
pN	pN(-)/pN(+)	0.005*	5.025	1.633-15.458
Number of pN(+)		0.057	1.295	0.992-1.691
Margin status	Clear/close or positive	0.861	1.111	0.342-3.612
Lymphatic invasion	(-)/(+)	0.014*	4.465	1.361-14.644
Vascular invasion	(-)/(+)	<0.001*	6.893	2.209-21.511
Perineural invasion	(-)/(+)	0.001*	6.229	2.018-19.215
ENE	pN(-)/ENE(-)/ENE(+)	0.002*	2.621	1.429-4.808
Local recurrence	(-)/(+)	0.004*	5.008	1.674-14.979

**Table 3:** Factors related with DSS (univariate analysis). ^*^P < 0.05. DSS, disease-specific survival; HR, hazard ratio; 95% CI, 95% confidence interval; PS, performance status; pN, pathological N; ENE, extranodal extension

Variable		*P*-value	HR	95% CI
Preoperative factor				
Age		0.289	0.971	0.919-1.026
Sex	Female/Male	0.501	0.621	0.155-2.485
PS	PS0/1/2/3	0.006*	2.996	1.364-6.579
Site	Gingiva/Hard palate	0.560	0.627	0.130-3.021
T stage	T1/2/3/4	0.127	6.301	0.592-67.051
N stage	N0/1/2	0.763	0.864	0.334-2.233
Differentiation	Well/Moderate	0.701	1.504	0.188-12.044
Mode of invasion	YK1/2/3/4	0.003*	5.808	1.806-18.678
Postoperative findings				
pN	pN(-)/pN(+)	0.003*	23.827	2.971-191.095
Number of pN(+)		0.010*	1.436	1.089-1.892
Margin status	Clear/close or positive	0.785	1.213	0.303-4.858
Lymphatic invasion	(-)/(+)	0.003*	7.651	2.033-28.794
Vascular invasion	(-)/(+)	0.001*	13.528	2.780-65.824
Perineural invasion	(-)/(+)	0.002*	12.823	2.650-62.047
ENE	pN(-)/ENE(-)/ENE(+)	<0.001*	4.635	2.035-10.559
Local recurrence	(-)/(+)	<0.001*	14.149	2.932-68.281

**Figure 2:**
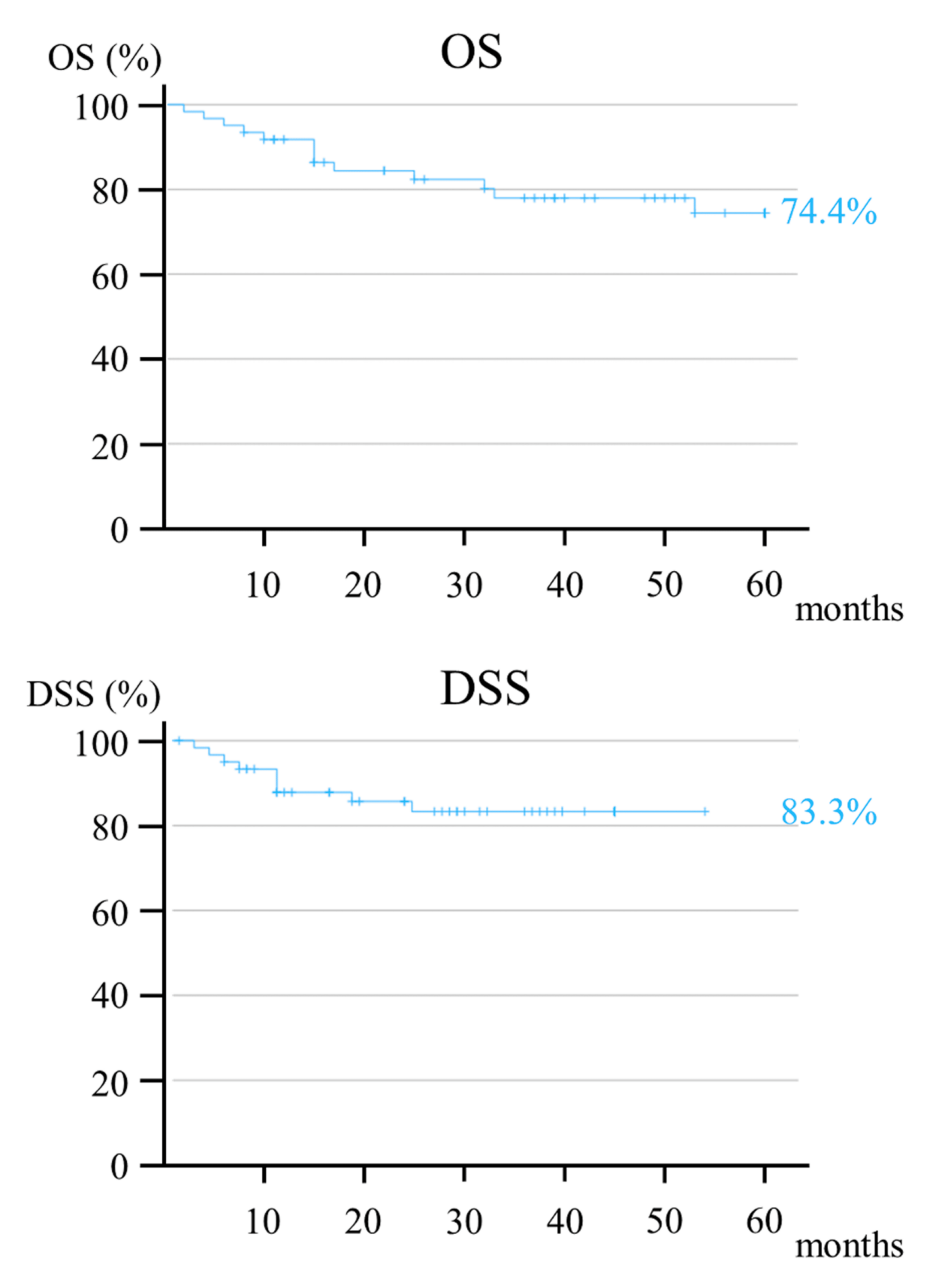
Kaplan-Meier curve showing the five-year OS and DSS. OS, overall survival; DSS, disease-specific survival

The five-year OS and DSS rates were 74.4% and 83.3%, respectively (Figure 2).

**Figure 3:**
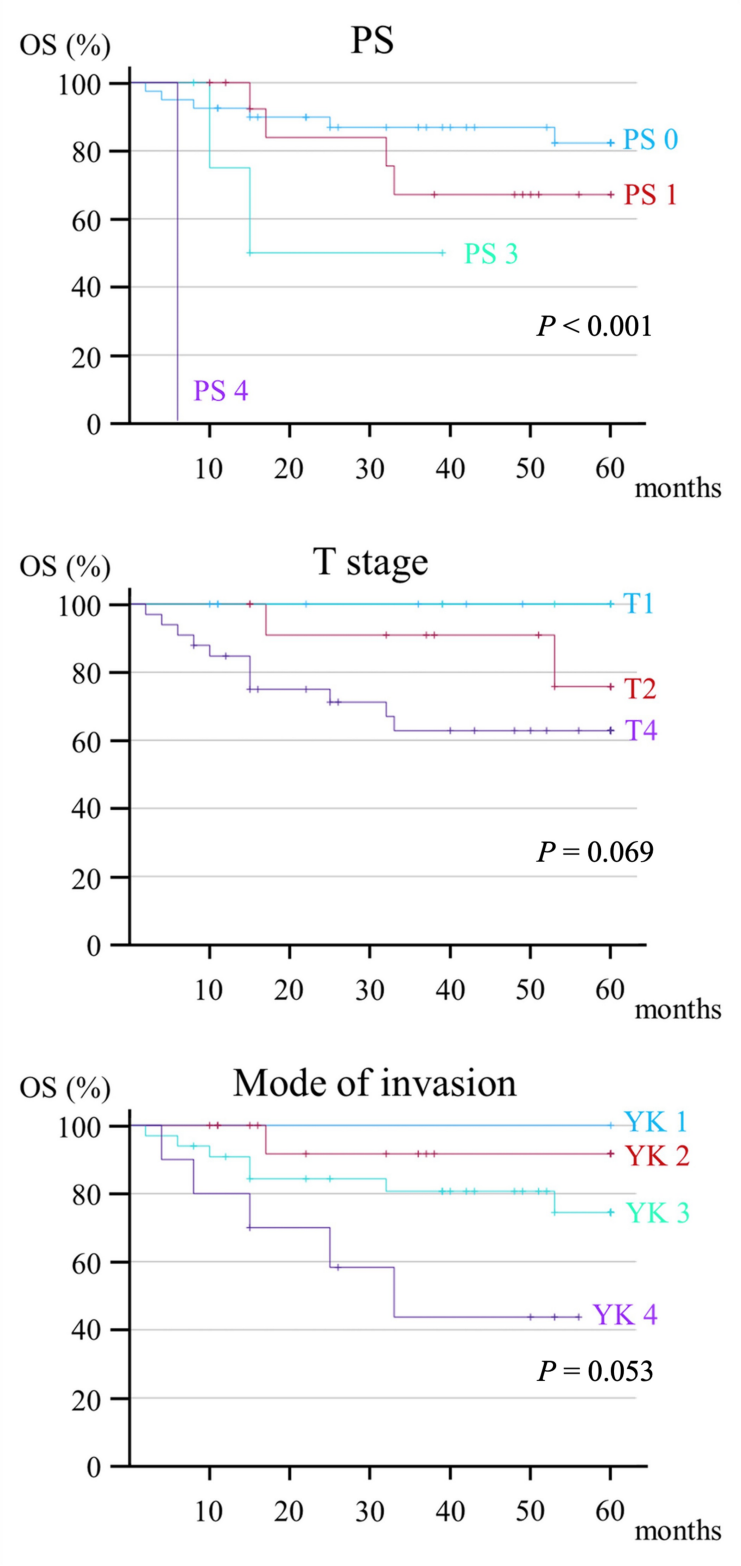
Kaplan-Meier curve of preoperative factors contributing to the five-year OS. OS, overall survival; PS, performance status

In univariate analysis, preoperative factors that significantly affected OS were PS (hazard ratio [HR], 2.623; *P* = 0.006), T stage (HR, 2.217; *P* = 0.031), and mode of invasion (HR, 3.047; *P* = 0.012) (Figure 3).

**Figure 4:**
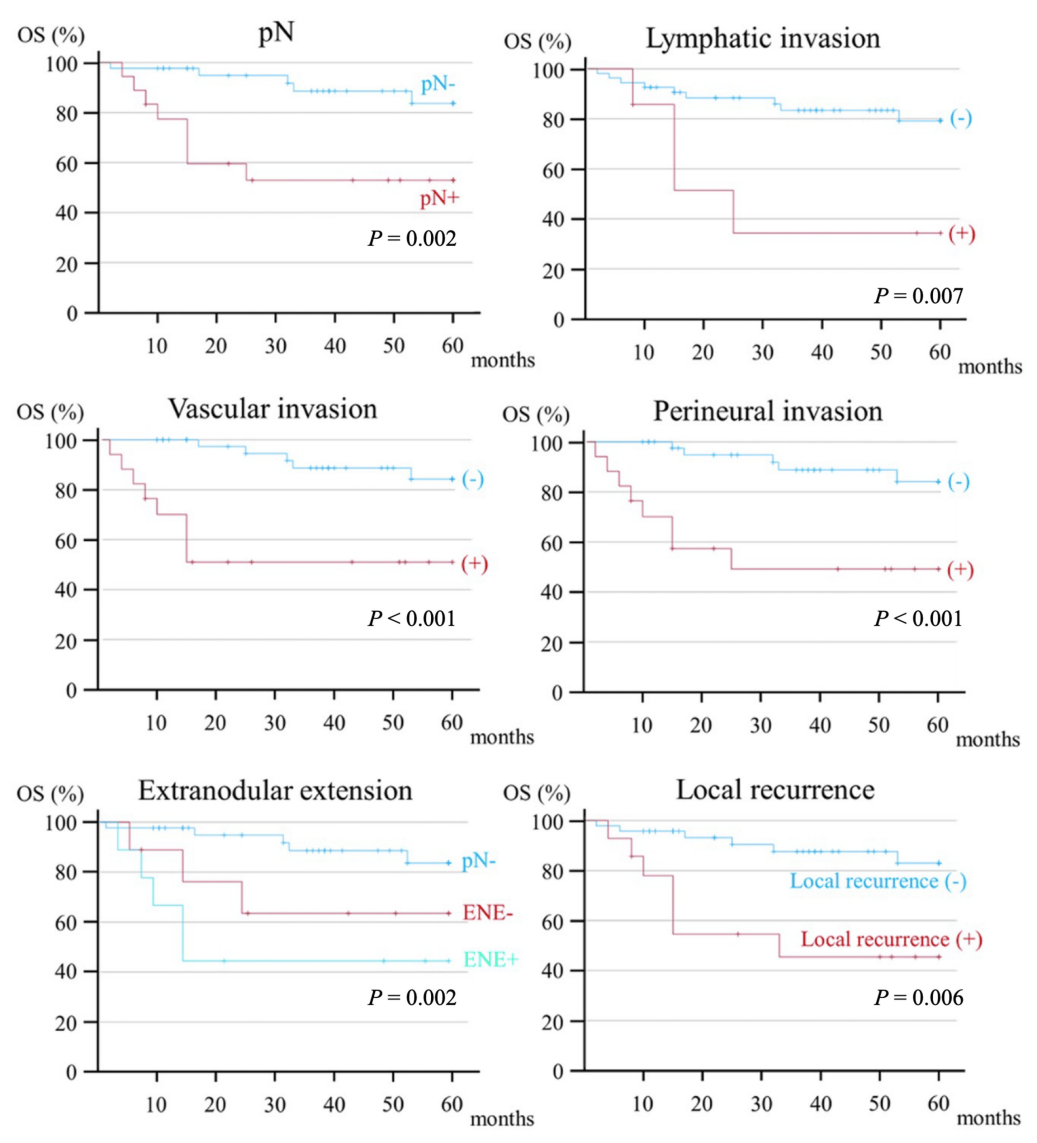
Kaplan-Meier curve of postoperative factors contributing to the five-year OS. OS, overall survival; pN, pathological N

Postoperative findings included pN (HR, 5.025; *P* = 0.005), lymphatic invasion (HR, 4.465; *P* = 0.014), vascular invasion (HR, 6.893; *P* < 0.001), and perineural invasion (HR, 6.893; *P* < 0.001), perineural invasion (HR, 6.229; *P* = 0.001), ENE (HR, 2.621; *P* = 0.002), and local recurrence (HR, 5.008; *P* = 0.004) were significantly associated with OS (Figure 4).

**Figure 5:**
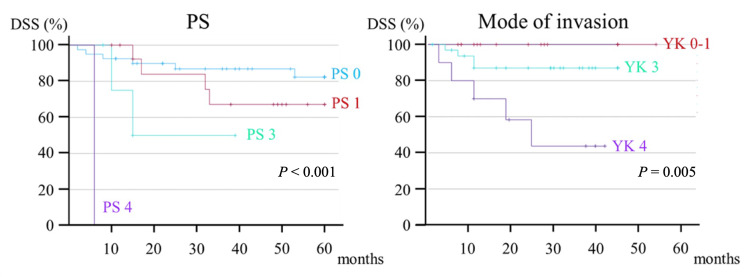
Kaplan-Meier curve of preoperative factors contributing to the five-year DSS. DSS, disease-specific survival; PS, performance status

In contrast, PS (HR, 2.996; *P* = 0.006) and mode of invasion (HR, 5.808; *P* = 0.003) were the preoperative factors that significantly affected DSS (Figure 5).

**Figure 6:**
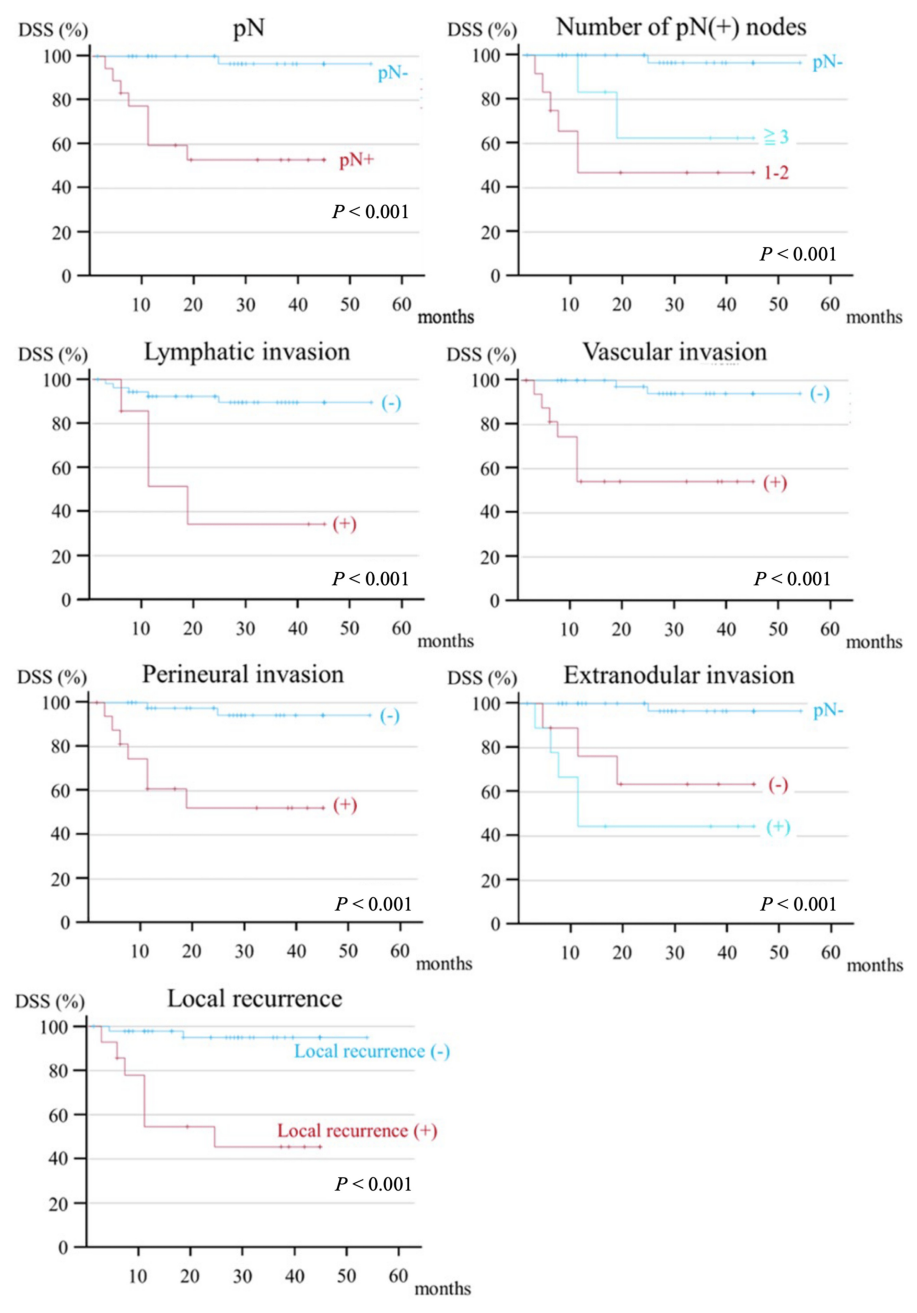
Kaplan-Meier curve of postoperative factors contributing to the five-year DSS. DSS, disease-specific survival; pN, pathological N

Postoperative findings included pN (HR: 23.827, *P* = 0.003), lymphatic invasion (HR, 7.651; *P* = 0.003), vascular invasion (HR, 13.528; *P* = 0.001), and perineural invasion (HR, 13.528; *P* = 0.001), perineural invasion (HR, 12.823; *P* = 0.002), ENE (HR, 4.635; *P* < 0.001), number of pN(+) (HR, 1.436; *P* = 0.010), and local recurrence (HR, 14.149; *P* < 0.001) were significantly associated with DSS (Figure 6).

In contrast, there was no significant correlation between margin status and prognosis.

Since local recurrence was one of the prognostic factors, it was investigated. Local recurrence was observed in 14 patients, confirmed between postoperative days 28 and 1373 (mean 281 days). Thirteen patients had T4a, and one patient had T4b. Factors significantly associated with local recurrence were T stage (HR, 6.666; *P* = 0.047) and mode of invasion (HR, 6.612; *P* < 0.001) as preoperative factors, including pN (HR, 8.222; *P* < 0.001), number of pN(+) (HR, 1.365; *P* = 0.010), lymphatic invasion (HR, 7.296; *P* < 0.001), vascular invasion (HR, 11.333; *P* < 0.001), perineural invasion (HR, 11.217; *P* < 0.001), and ENE (HR, 9.145; *P* < 0.001). However, there was no correlation between margin status and local recurrence (Table 4).

**Table 4:** Factors related to local control (univariate analysis). ^*^P < 0.05. HR, hazard ratio; 95% CI, 95% confidence interval; PS, performance status; pN, pathological N; ENE, extranodal extension

Variable		*P*-value	HR	95% CI
Preoperative factor				
Age		0.451	0.982	0.938-1.029
Sex	Female/Male	0.075	0.313	0.087-1.125
PS	PS0/1/2/3	0.051	1.897	0.998-3.609
Site	Gingiva/Hard palate	0.351	2.626	0.344-20.209
T stage	T1 / 2 / 3 / 4	0.047*	6.666	1.029-43.198
N stage	N0/1/2	0.176	1.519	0.829-2.784
Differentiation	Well/Moderate	0.982	0.976	0.128-7.473
Mode of invasion	YK1/2/3/4	<0.001*	6.612	2.566-17.033
Postoperative findings				
pN	pN(-)/pN(+)	<0.001*	8.222	2.562-26.383
Number of pN(+)		0.010*	1.365	1.089-1.728
Margin status	Clear/close or positive	0.501	1.456	0.487-4.349
Lymphatic invasion	(-)/(+)	<0.001*	7.296	2.394-22.236
Vascular invasion	(-)/(+)	<0.001*	11.333	3.486-36.843
Perineural invasion	(-)/(+)	<0.001*	11.217	3.461-36.353
Extranodal extension	pN(-)/ENE(-)/ENE(+)	<0.001*	9.145	3.172-26.368

## Discussion

In this study, we investigated the prognostic factors for SCC of the maxillary gingiva and hard palate. Clinical disease progression such as T and pN; histological factors such as mode of invasion, lymphatic invasion, vascular invasion; and perineural invasion were closely related to prognosis. Moreover, local recurrence was significantly associated with the prognosis. In contrast, margin status did not significantly affect OS, DSS, and local recurrence.

Some previous clinical studies have demonstrated that not only the degree of progression of the primary tumor but also the histological grade or prevalence of cervical lymph node metastasis are associated with prognosis in oral cancer. Yamamoto et al. reported that the mode of invasion was associated with bleomycin efficacy and prognosis [12], and Umeda et al. reported that histological features were associated with the prevalence of cervical lymph node metastasis [13]. Hasegawa et al. demonstrated that the presence of cervical lymph node metastasis to Level IV/V significantly decreased the three-year cumulative survival rate in multi-institutional research of 669 pN+ cases [14], and Larsen et al. also reported that cervical lymph node metastasis with ENE significantly reduced the five-year DSS rate [15]. Furthermore, histological grade and tumor budding have been confirmed as prognostic factors for early-stage oral SCC [16,17]. It has also been reported that close or positive margins are associated with local recurrence. In a multi-institutional joint research of 564 cases of stages I and II tongue cancer, Otsuru et al. demonstrated that resection margins of less than 3.3 mm for horizontal and less than 3.1 mm for vertical margins were significantly associated with local recurrence, although these statuses were not associated with prognosis [18].

However, the present study revealed that the margin status did not correlate with either local recurrence or survival rate. This is not only because the structure of the maxilla is complex and it is difficult to identify the surgical margin three-dimensionally from the resected specimen, but also because cancer that extends into the masticator space or sinus cavity might have tumor satellites beyond the resected margin, even if the margin status is negative. Previous reports have indicated that patients with tumor satellites exhibit a higher incidence of perineural invasion (70.6% vs. 32.1%, *P* = 0.002) and lymphovascular invasion (29.4% vs. 9.8%, *P* = 0.022), suggesting a more locally aggressive disease course. However, tumor satellites may be overlooked in routine pathological evaluations, highlighting the need for standardized diagnostic criteria and careful histopathological assessment [19]. Also, in this study, local recurrence was observed in patients with lymphatic, vascular, and perineural invasion, regardless of margin status, suggesting that attention should be paid when cases with negative surgical margins and high histological grade are encountered.

As maxillary gingival carcinoma is one of the least common oral cancers, there are few reports of clinicopathological research based on a large number of cases. Regarding prognosis, Cheval et al. reported a five-year OS of 57.3% in 123 patients [20], and Yang et al. reported 57.3% [21]. Cheval et al. reported on prognostic factors, perineural invasion, tumor size, bone invasion, pT classification, and pN classification [20]. However, Yang et al. reported that differentiation, T classification, margin status, cervical metastasis, and local recurrence were prognostic factors [21]. Various reports have been made on the treatment of cervical lymph node metastasis in maxillary gingival cancer. Mourouzis et al. recommended END for maxillary gingival cancer in a study of 17 cases [22]. Dalal and McLennan recommended END for 30 T4 cases [23], Feng et al. for 129 T2-T4 cases [24], and Qu et al. for 107 T3-T4 cases [5]. In addition, the systematic review by Zhang and Peng and de la Fuente et al. recommended END for T3-4 cases [25,26]. In contrast, Park et al. reported that the frequency of occult cervical metastasis was not sufficiently high to recommend END, and local control of the primary tumor is more important in maxillary gingival cancer [4]. Hence, there were few maxillary gingival carcinoma cases and the treatment strategy remains controversial.

In our study, the five-year OS and DSS rates were 74.4% and 83.3%, respectively, better than those previously reported. END is not routinely performed in our department, and most of the 14 patients with poor prognosis (12 out of 14) had T factors, and only two (3.3%) had N factors, suggesting that END might not always be necessary for maxillary gingival cancer and that primary tumor control would be more important, as demonstrated by Park et al. [4]. Several factors may have contributed to the high survival rates observed in this study. First, 90% of the patients had an ECOG-PS of 0-1, indicating good overall health, which likely enabled them to receive appropriate standard treatment. Second, regarding the mode of invasion, 85% of the patients were classified as YK1-YK3, suggesting lower tumor aggressiveness and a reduced risk of lymph node metastasis. Additionally, high-precision imaging modalities, such as CT, MRI, and PET-CT, may have facilitated the accurate assessment of occult metastases, contributing to appropriate treatment decisions. Furthermore, a meticulous follow-up system may have allowed for the early detection of lymph node metastases and timely therapeutic intervention, potentially improving survival outcomes.

Thirty-three of the 61 (54%) cases in this study consisted of advanced T4 (T4a, 31 cases; T4b, 2 cases). Local recurrence was observed in 14 cases (23.0%), all of which were T4 cases, and there was no local recurrence in T1-3 cases. The surgical approach for T4 cases is based on a subtotal maxillectomy in which the bone of the orbital floor is preserved. When cancer is recognized in the masticator space and diagnosed as T4b, masticatory space dissection, including the pterygoid muscle, is applied in combination with subtotal maxillectomy [8].

Cases diagnosed as T4a may include cases of maxillary sinus invasion along the maxillary sinus mucosa beyond the imaging range of CT and MRI and cases diagnosed as T4a without invasion of the masticatory space or pterygoid process according to preoperative assessment but with microscopic involvement of the masticatory space. Most local recurrence cases had a high histological grade with lymphatic, vascular, or perineural invasion. Therefore, it is necessary to consider the indication of postoperative CRT for patients with T4a and a high histological grade, in addition to imaging diagnosis, regardless of the margin status.

This study had several limitations. First, because maxillary gingival SCC occurs infrequently among oral cancers, this study had a limited sample size (61 cases), which restricted the statistical power and prevented multivariate analysis. Furthermore, the opt-out method has the potential to introduce selection bias by disproportionately including specific populations while inadvertently excluding others. Consequently, a comprehensive evaluation of poor prognostic factors was not feasible. Second, the number of patients who received postoperative therapy was small, making it difficult to assess its effectiveness. Additionally, while this study provided evidence that histological grade is an important prognostic factor, no significant correlation between margin status and local recurrence or prognosis was observed.

Although the follow-up period in this study was sufficient to assess long-term outcomes, the limited sample size underscores the necessity for further research. Future studies should aim to validate these findings by incorporating a larger cohort and conducting multi-institutional collaborative research to enhance the generalizability and robustness of the results.

## Conclusions

This study analyzed 61 cases of SCC of the maxillary gingiva or hard palate clinicopathologically, reporting five-year OS and DSS rates of 74.4% and 83.3%, respectively. Among these cases, 14 exhibited uncontrolled disease, all of which were classified as cT4 tumors. Notably, 12 of these cases had poor local control of the primary tumor. In contrast, no cT1-T3 cases experienced local recurrence following standard treatment. Both tumor stage and histological factors, such as mode of invasion, lymphatic invasion, vascular invasion, and perineural invasion, were identified as poor prognostic factors. However, margin status was not significantly associated with local recurrence or overall survival. This finding might be attributed to the presence of tumor satellites, which could contribute to tumor progression despite negative surgical margins. These results suggest that the standard treatment approach for T4 cases and those with high-grade histological malignancy should be reconsidered to improve clinical outcomes.
